# Polarizable AMOEBA force field predicts thin and dense hydration layer around monosaccharides†

**DOI:** 10.1039/d4cc04415k

**Published:** 2024-11-12

**Authors:** Luke A. Newman, Mackenzie G. Patton, Breyanna A. Rodriguez, Ethan W. Sumner, Valerie Vaissier Welborn

**Affiliations:** a Department of Chemistry, Virginia Tech Blacksburg VA 24061 USA vwelborn@vt.edu; b Macromolecules Innovation Institute, Virginia Tech Blacksburg VA 24061 USA; c Department of Chemistry and Biochemistry, Bridgewater College Bridgewater VA 22812 USA; d Department of Chemistry and Biochemistry, Old Dominion University Norfolk VA 23529 USA

## Abstract

Polarizable force fields crucially enhance the modeling of macromolecules in polar media. Here, we present new parameters to model six common monosaccharides with the polarizable AMOEBA force field. These parameters yield a thinner, but denser, hydration layer than that previously reported. This denser hydration layer results in eliminating non-physical aggregation of glucose in water—an issue that has plagued molecular dynamics simulations of carbohydrates for decades.

## Abstract 2

Polarizable force fields improve the modeling of carbohydrates in polar media.

## Graphical abstract

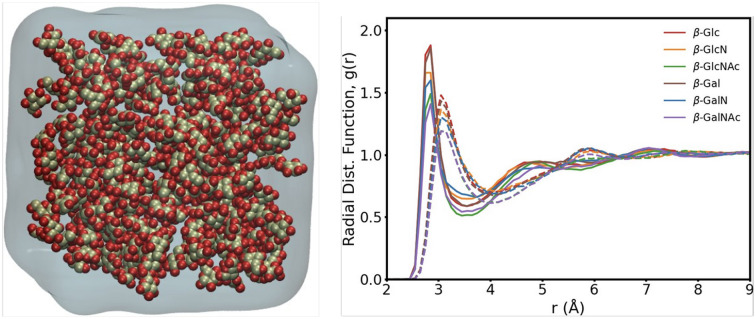

Ubiquitous in Nature, monosaccharides have become building blocks for novel molecules and materials.^1–3^ A vast set of monosaccharides with differing numbers and types of functional groups exist. Further, these building blocks form a vast array of polysaccharides, glycoproteins, glycolipids or glycomolecules. This modularity makes carbohydrates relevant to many research fields. However, it also hinders our ability to fully realize, predict and control the properties of natural and synthetic molecules containing carbohydrates. Characterizing these molecules with solution nuclear magnetic resonance, X-ray and neutron spectroscopies provides microscopic, ensemble properties, which can be hard to interpret at the molecular level.^4–7^ Modeling can inform these properties, providing that the level of theory used accounts for the intermolecular interactions that govern carbohydrate properties. In practice, carbohydrate modeling gravitates towards classical molecular dynamics (MD), whose accuracy depends on that of the underlying force field.^1,2,8,9^ Over the past decade, multiple force fields have been tested, or developed specifically, for carbohydrate modeling, including, but not limited to, GROMOS, CHARMM, Drude (polarizable), GLYCAM, and CSFF.^8–11^

Previous MD studies highlight the importance of polarization for the modeling of monosaccharides in water.^8,9^ Indeed, explicit polarization shows an increase in conformational flexibility, expansion of monosaccharides' solvation shell, and changes in distance and orientation of proximal waters.^9^ Meanwhile, implicit water polarization (*e.g.*, TIP5P) better balances the van der Waals attraction term between and among carbohydrates. More specifically, force fields that use the 12–6 Lennard-Jones potential overestimate the attraction between monosaccharides, yielding to non-physical aggregation of β-d-glucose at low concentration.^8,12^ Although they demonstrated that the issue can be mitigated by improving the electrostatic term with polarization and re-optimizing the Lennard-Jones parameters,^8,13^ non-physical aggregation remains a challenge to predictive models of carbohydrates in diverse environments.

Here, we assessed the AMOEBA polarizable force field^14^ for modeling carbohydrates in solution. AMOEBA includes explicit polarization for water and carbohydrates *via* induced atomic dipoles, as well as a buffered 14–7 Halgren potential for van der Waals interactions, which addresses the limitations of other force fields listed above. We used poltype 2^15^ as is (*i.e.*, without refining the generic van der Waals or torsion parameters), to compute new AMOEBA parameters for the α and β anomers of d-glucose (Glc), d-glucosamine (GlcN), *N*-acetyl-d-glucosamine (GlcNAc), d-galactose (Gal), d-galactosamine (GalN), and *N*-acetyl-d-galactosamine (GalNAc), shown in Fig. 1.

**Fig. 1:**
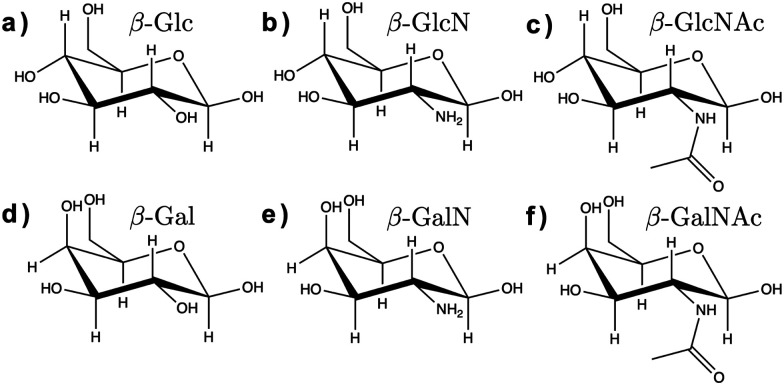
Chemical structure of the β anomers of the six monosaccharides studied here. (a) Glc, (b) GlcN, (c) GlcNAc, (d) Gal, (e) GalN, (f) GalNAc. The chemical structure of the *α* anomers is provided in ESI† (Fig. S1).

Using the new poltype 2 AMOEBA parameters, we performed an energy minimization, followed by 150 ns MD simulation of each monosaccharide in water with constant number of particles, pressure and temperature (NPT) in Tinker 9.^16^ The first 5 ns of the MD were used as equilibration and the remaining 145 ns as production run to compute the properties described below. Each simulation was run twice, once with mutual polarization, converging induced dipoles at each step within 10^−5^ D, and once without the dipole polarization term (see input files in ESI†).

Average bond lengths, angles, and ring dihedrals (ESI†) agree with experimental data for all twelve anomers. Comparing these geometric variables with and without mutual polarization, we see that polarization does not change the structure of the monosaccharides, nor does it yield enhanced flexibility. This suggests that force field accuracy is mainly revealed in the solution properties of the modeled carbohydrates (*i.e.*, the structure and density of the hydration layer, diffusivity, *etc.*), which will be the focus of this paper. Nevertheless, we note two observations about the conformation of these monosaccharides, as predicted with AMOEBA.

First, we record ring puckering for a majority of the α anomers. This contrasts with the β anomers that all remain in a stable chair conformation throughout the duration of the simulation. In Fig. 2, we show the evolution of the ring dihedral C_1_–C_2_–C_3_–C_4_ for α-Glc. We see that α-Glc spends a majority of the simulation in the chair conformation (C_1_–C_2_–C_3_–C_4_∼–55°) but also temporarily visits alternative ring puckered conformations. Overall, α-Glc, α-GlcN, α-GlcNAc, α-Gal, α-GalN and α-GalNAc spends 2.9, 7.4, 0.09, 0.07, 2.6 and 0.05% of the 145 ns MD trajectory, respectively, in a conformation other than chair. Extensions of the simulations of α- and β-Glc, GlcN, Gal and GalN to 300 ns, and replicate simulations (ESI†), confirm this trend. Although this effect has been reported before,^17,18^ it is non trivial to validate against experiments and it remains possible that these conformational switches are non-physical. However, we obtain similar results when using the β AMOEBA parameters to model the α anomers (Fig. S4–S7, ESI†), suggesting that this effect is due to the structure and not the force field parameters.

**Fig. 2:**
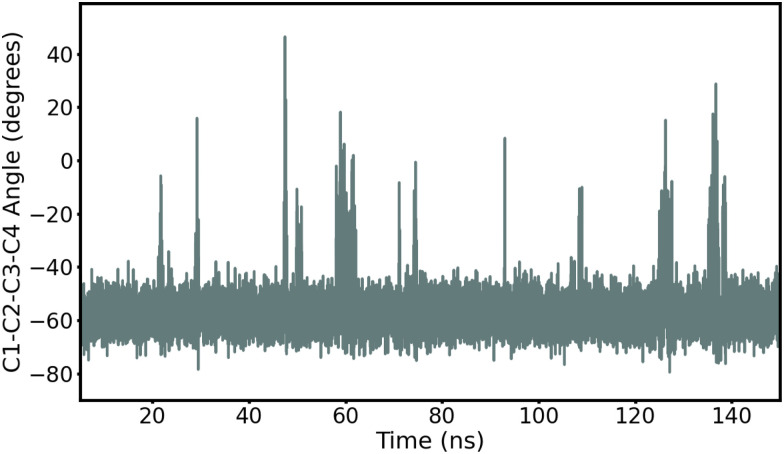
Time evolution of the torsion angle C_1_–C_2_–C_3_–C_4_ in α-Glc during the 145 ns production MD with AMOEBA (mutual polarization). C_1_–C_2_–C_3_–C_4_ is −55° when α-Glc is in chair conformation.

Second, the dihedral O_6_–C_6_–C_5_–O_5_ reveals a ratio for the three gg, gt, and tg populations that do not agree with experimental data. For example, we find that β-Glc is 70(39)% gg, 28(22)% gt and 2(39)% tg in our simulations with mutual (no) polarization, compared with 31, 61 and 8% in experiments.^4,5^ This likely indicates that this degree of freedom equilibrates on timescales longer than is simulated here. A solution would be to separate the frames that characterize each rotamer, compute their properties independently and reweigh them according to the experimental ratio. However, we do not anticipate the different rotamers to have significantly different solution properties. Therefore, for the remainder of this paper, the data are computed from the unmodified MD trajectories.

In Fig. 3, we present the radial distribution function of the hydroxyl oxygens to the water oxygens for the β anomers (see Fig. S2 for the α anomers plot, ESI†). In Table 1, we show the position of the first peak, position of the first minima and coordination number at the first minima, for β-Glc, as reference.

**Fig. 3:**
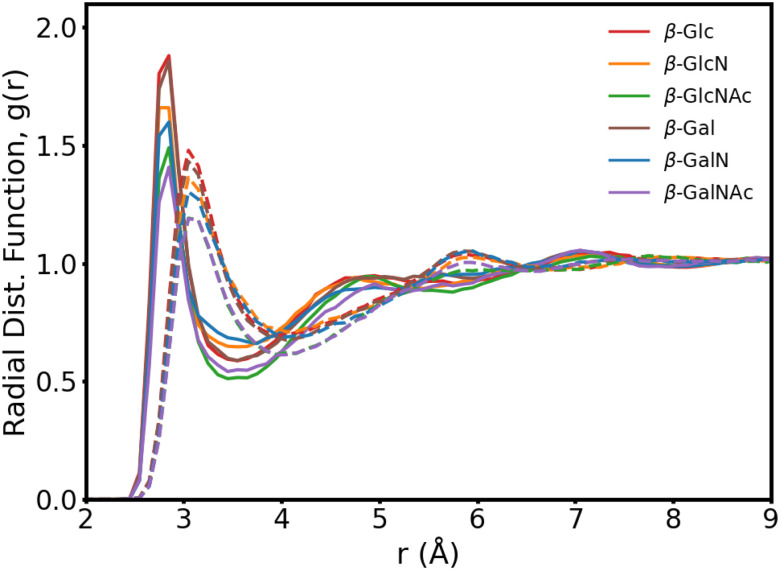
Carbohydrate–water radial distribution function for the six β anomers, with (solid lines) and without (dotted lines) mutual polarization. Each curve is an average of the O_1_–O_w_, O_2_/N_2_–O_w_, O_3_–O_w_ and O_4_–O_w_ distribution functions, where O_w_ is the water oxygen.

**Table 1:** First peak position (*r*_0_), first minimum position (*r*_min_) and coordination number (CN) for hydroxyl-water radial distribution function of β-Glc with and without mutual polarization

Oxygen	Mutual pol.			No pol.		
	*r*_0_ (Å)	*r*_min_ (Å)	CN	*r*_0_ (Å)	*r*_min_ (Å)	CN
O_1_	2.85	3.55	4.16	3.05	4.25	6.18
O_2_	2.85	3.45	3.59	3.05	4.05	4.94
O_3_	2.85	3.65	4.22	3.05	3.95	4.48
O_4_	2.85	3.45	3.37	3.05	3.95	4.21
Average	2.85	3.55	3.90	3.05	4.05	4.92
O_6_	2.85	3.35	3.31	3.05	3.85	4.28

The six monosaccharides exhibit a very similar hydration pattern with water oxygens, most likely, 2.85 Å away from the hydroxyl oxygens (Table 1). Looking at the radial distribution functions from our MD simulations performed without polarization (dotted lines in Fig. 3), we see shorter and broader peaks. These results indicate that polarization enhances the structure of proximal waters, but does not result in an expanded hydration shell as reported elsewhere.^9^ Rather, we compute an hydration shell of 3.5 Å for β-Glc, compared to 4.05 Å without polarization (Tables 1), and 3.8 Å as predicted by the polarizable Drude force field.^9^ In our simulations, we see a tighter, more ordered, hydration layer with higher coordination number, corresponding to a significantly denser hydration shell than has been previously observed with other force fields.^9^

In Table 2, we list the hydration number of each monomer, computed with and without mutual polarization, as well as the number of acceptor and donor hydrogen bonds (H-bonds) to the hydroxyls and nitrogen groups. We find that Glc is surrounded by 25 ± 8 water molecules, which is consistent with the 21 hydration number found with dielectric spectroscopy.^19^ Further, we observe a relatively large standard deviation of the hydration number for all monosaccharides, regardless of the anomeric form. This suggests a dynamic hydration layer where the water molecules exchange position on a timescale of tens of picoseconds. Interestingly, we see that polarization increases the hydration number by about 5 in all cases and the standard deviation by 2. Therefore, polarization induces a thin, ordered, hydration layer that is also very dynamic. Finally, we see that the hydroxyl groups in all monosaccharides are better hydrogen bond acceptors than they are donors, consistent with previous work.^11^

**Table 2:** Hydration number (number of water molecules within 4 Å of each monosaccharide) and number of acceptor and donor hydrogen bonds to the hydroxyl groups

System	Hydration number	Acceptor H-bonds	Donor H-bonds
α-Glc (mutual pol.)	24.7 ± 7.3	5.4 ± 1.3	3.6 ± 1.0
α-Glc (no pol.)	19.4 ± 5.9	2.9 ± 1.3	1.7 ± 1.0
β-Glc (mutual pol.)	24.6 ± 7.6	5.5 ± 1.4	3.5 ± 1.0
β-Glc (no pol.)	19.4 ± 5.9	2.7 ± 1.3	1.6 ± 1.0
α-GlcN (mutual pol.)	24.5 ± 7.3	5.3 ± 1.2	3.2 ± 1.0
α-GlcN (no pol.)	19.0 ± 5.8	3.0 ± 1.3	1.5 ± 1.0
β-GlcN (mutual pol.)	24.3 ± 7.4	5.4 ± 1.3	3.4 ± 1.1
β-GlcN (no pol.)	19.5 ± 5.9	3.0 ± 1.3	1.6 ± 1.0
α-GlcNAc (mutual pol.)	28.6 ± 8.3	4.5 ± 1.2	3.2 ± 1.0
α-GalNAc (no pol.)	22.3 ± 7.0	2.2 ± 1.2	1.7 ± 1.1
β-GlcNAc (mutual pol.)	28.0 ± 8.8	4.6 ± 1.3	3.6 ± 1.1
β-GlcNAc (no pol.)	22.5 ± 6.8	2.3 ± 1.2	1.8 ± 1.0
α-Gal (mutual pol.)	25.3 ± 7.3	5.2 ± 1.3	3.5 ± 1.0
α-Gal (no pol.)	19.6 ± 5.9	2.8 ± 1.3	1.6 ± 1.0
β-Gal (mutual pol.)	24.4 ± 7.4	5.3 ± 1.3	3.4 ± 1.0
β-Gal (no pol.)	19.6 ± 5.9	2.8 ± 1.3	1.6 ± 1.0
α-GalN (mutual pol.)	24.0 ± 7.4	5.0 ± 1.2	3.1 ± 1.0
α-GalN (no pol.)	19.3 ± 6.0	3.0 ± 1.3	1.6 ± 1.0
β-GalN (mutual pol.)	25.2 ± 7.3	5.3 ± 1.3	3.3 ± 1.1
β-GalN (no pol.)	19.6 ± 5.9	2.9 ± 1.3	1.6 ± 1.0
α-GalNAc (mutual pol.)	28.2 ± 8.7	4.3 ± 1.2	3.3 ± 1.0
α-GalNAc (no pol.)	22.6 ± 6.9	2.3 ± 1.2	1.6 ± 1.0
β-GalNAc (mutual pol.)	27.6 ± 8.7	4.4 ± 1.2	3.4 ± 1.0
β-GalNAc (no pol.)	22.7 ± 6.9	2.4 ± 1.2	1.7 ± 1.0

To further test the performance of these new AMOEBA force field parameters for monosaccharides, we ran MD simulations (150 ns in the NPT ensemble) of multiple β-Glc in water. Following previous work,^8^ we simulated six different concentrations, all below the concentration at which β-Glc aggregates: 0.25, 0.5, 1, 2, 3 and 4 mol kg^−1^. In each case, we computed the radial distribution function of the center-of-mass (COM) of β-Glc, as well as their mean-square displacement. We fitted the mean-square displacement as a function of time with a linear function (Fig. S3, ESI†) to extract the diffusion coefficient for each concentration:1
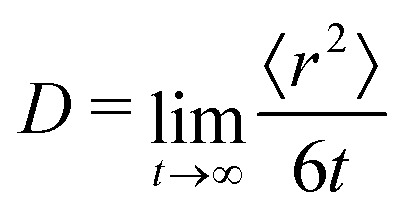
where *D* is the diffusion coefficient, 〈*r*^2^〉 the mean-square displacement of β-Glc (the mean is carried over the number of β-Glc in the simulation) and *t* the time. Fig. 4 presents the results. In previous MD studies, non-physical β-Glc aggregation manifested within a few ns, characterized by a sharp peak in the radial distribution function at about 5.5 Å. In our simulations, we do not see this feature (Fig. 4c). Rather, we see a much smaller and broader peak at 7 Å, whose integration (Fig. 4d) scales with the concentration of β-Glc. This suggests that non-physical aggregation of the monosaccharides in water does not equate to the broad peak in our radial distribution function. Further, the diffusion coefficients computed from these simulations agree well with experimental data (Fig. 4b), where AMOEBA only slightly underestimates the diffusion of β-Glc in water, outperforming other force fields.^8^ Running MD for the highest concentration (*c* = 4 mol kg^−1^) without mutual polarization results in a similar, although slightly broader, COM radial distribution function (light green curve in Fig. 4c and d). This result emphasizes van der Waals interactions as governing the aggregation of monosaccharides in solution, not polarization.

**Fig. 4:**
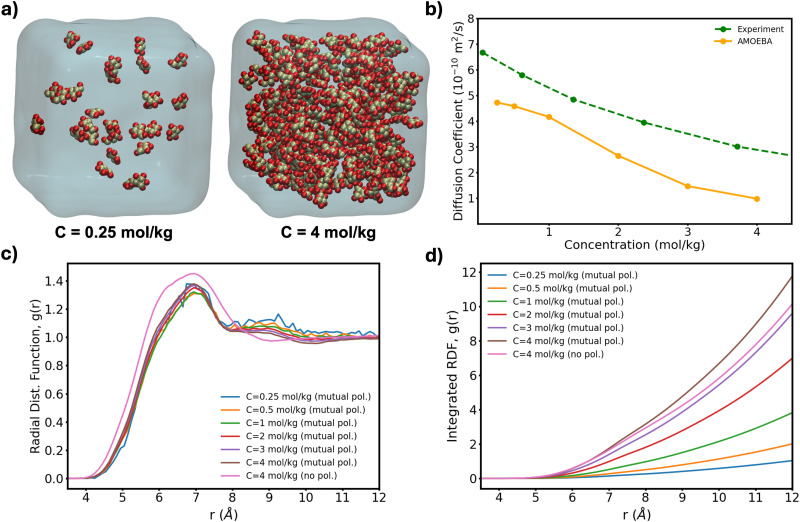
(a) Lowest and highest Glc concentration modeled here. (b) Diffusion coefficient computed from eqn (1) as a function of concentration for our box sizes of ∼60^3^ Å^3^ (∼20 k atoms). Extrapolation of MD-calculated diffusion coefficients to bulk conditions can yield a 10% increase^20^ although no correction has been applied here. (c) Radial distribution function of the COM of β-Glc and (d) corresponding integration data.

In summary, the polarizable AMOEBA force field with its robust multipole expansion, atomic induced dipoles and buffered 14–7 Halgren potential outperforms other force fields when modeling monosaccharides in solution. As AMOEBA has also been proven accurate for water properties and protein modeling, it provides the opportunity for a unified set of parameters that can described saccharides in diverse hydrated environments.

The authors thank GlycoMIP, a National Science Foundation Materials Innovation Platform funded through Cooperative Agreement DMR-1933525; GlycoTREE, a National Science Foundation and US Department of Defense Research Experiences for Undergraduates grant DMR-2244483; the American Chemical Society Petroleum Research Fund under award number 66767-ND6 and the National Institute of Health, National Institute of General Medical Sciences, under award number R35-GM150409 for financial support.

## Data availability

The data supporting this article have been included as part of the ESI.† Input files, including Poltype 2 and Tinker 9 input with parameters files are available at https://github.com/WelbornGroup.

## Conflicts of interest

There are no conflicts to declare.

## Supplementary Material

CC-060-D4CC04415K-s001
