# Supplementary material for: Polarizable AMOEBA force field predicts thin and dense hydration layer around monosaccharides
Source: Chem Commun (Camb). 2024 Nov 12;60(97):14431–4. doi: 10.1039/d4cc04415k (PMC11566667; doi:10.1039/d4cc04415k)
Supplement: CC-060-D4CC04415K-s001 [file CC-060-D4CC04415K-s001.pdf]

Supplementary Information (SI) for ChemComm.
This journal is © The Royal Society of Chemistry 2024

Polarizable AMOEBA force field predicts thin and dense hydration layer around monosaccharides.

Luke A. Newman, Mackenzie Patton, Breyanna Rodriguez, Ethan Sumner, and Valerie Vaissier Welborn*

E-mail: vwelborn@vt.edu

MD input file availability

Poltype 2 and Tinker 9 input with parameters files are available at https://github.com/WelbornGroup/.

Average bond, angle and dihedrals for monosaccharides in water

Table 1: Bond lengths reported in Å as averages over 145 ns of MD in the NPT ensemble (after 5 ns equilibration). The error is the standard deviation.

	β-Glc		β-GlcN		β-GlcNAc		Exp
Bond	mutual pol.	no pol.	mutual pol.	no pol.	mutual pol.	no pol	
C1-C2	1.54 ± 0.04	1.53 ± 0.03	1.53 ± 0.04	1.53 ± 0.04	1.56 ± 0.04	1.56 ± 0.04	1.52
C2-C3	1.55 ± 0.04	1.54 ± 0.03	1.56 ± 0.04	1.56 ± 0.04	1.53 ± 0.04	1.53 ± 0.04	1.53
C3-C4	1.55 ± 0.04	1.55 ± 0.03	1.53 ± 0.04	1.53 ± 0.04	1.56 ± 0.04	1.56 ± 0.04	1.52
C4-C5	1.57 ± 0.04	1.57 ± 0.03	1.56 ± 0.04	1.57 ± 0.04	1.56 ± 0.04	1.56 ± 0.04	1.53
C5-C6	1.54 ± 0.04	1.53 ± 0.04	1.54 ± 0.04	1.55 ± 0.04	1.54 ± 0.04	1.55 ± 0.04	1.51
C1-O1	1.41 ± 0.03	1.41 ± 0.03	1.42 ± 0.03	1.42 ± 0.03	1.40 ± 0.03	1.40 ± 0.03	1.40
C2-O2	1.45 ± 0.04	1.44 ± 0.03	1.48 ± 0.03	1.49 ± 0.03	1.47 ± 0.03	1.47 ± 0.03	1.43
C3-O3	1.45 ± 0.04	1.43 ± 0.03	1.47 ± 0.04	1.46 ± 0.04	1.45 ± 0.04	1.45 ± 0.04	1.43

C4-O4	1.45 ± 0.04	1.44 ± 0.03	1.45 ± 0.04	1.45 ± 0.04	1.46 ± 0.04	1.46 ± 0.04	1.43
C5-O5	1.44 ± 0.04	1.46 ± 0.05	1.44 ± 0.04	1.44 ± 0.04	1.46 ± 0.04	1.46 ± 0.04	1.45
C6-O6	1.45 ± 0.04	1.43 ± 0.03	1.45 ± 0.04	1.44 ± 0.04	1.44 ± 0.04	1.44 ± 0.04	1.42
O5-C1	1.45 ± 0.04	1.43 ± 0.04	1.45 ± 0.04	1.45 ± 0.04	1.46 ± 0.04	1.46 ± 0.04	1.43

Table 2: Bond lengths reported in Å as averages over 145 ns of MD in the NPT ensemble (after 5 ns equilibration). The error is the standard deviation.

	β-Gal		β-GalN		β-GalNAc		Exp
Bond	mutual pol.	no pol.	mutual pol.	no pol.	mutual pol.	no pol	
C1-C2	1.54 ± 0.04	1.55 ± 0.04	1.53 ± 0.04	1.55 ± 0.04	1.56 ± 0.04	1.57 ± 0.04	1.52
C2-C3	1.55 ± 0.04	1.55 ± 0.04	1.56 ± 0.04	1.54 ± 0.04	1.54 ± 0.04	1.54 ± 0.04	1.53
C3-C4	1.56 ± 0.04	1.57 ± 0.04	1.53 ± 0.04	1.57 ± 0.04	1.56 ± 0.04	1.57 ± 0.04	1.52
C4-C5	1.56 ± 0.04	1.56 ± 0.04	1.56 ± 0.04	1.53 ± 0.04	1.55 ± 0.04	1.55 ± 0.04	1.53
C5-C6	1.54 ± 0.04	1.55 ± 0.04	1.54 ± 0.04	1.55 ± 0.04	1.55 ± 0.04	1.55 ± 0.04	1.51
C1-O1	1.41 ± 0.03	1.41 ± 0.03	1.42 ± 0.03	1.41 ± 0.03	1.40 ± 0.03	1.41 ± 0.03	1.40
C2-O2	1.45 ± 0.04	1.45 ± 0.04	1.48 ± 0.03	1.48 ± 0.03	1.47 ± 0.04	1.47 ± 0.03	1.43
C3-O3	1.45 ± 0.04	1.44 ± 0.04	1.47 ± 0.04	1.44 ± 0.04	1.45 ± 0.04	1.45 ± 0.04	1.43
C4-O4	1.45 ± 0.04	1.45 ± 0.04	1.45 ± 0.04	1.45 ± 0.04	1.45 ± 0.04	1.45 ± 0.04	1.43
C5-O5	1.44 ± 0.04	1.44 ± 0.04	1.44 ± 0.04	1.43 ± 0.04	1.43 ± 0.04	1.43 ± 0.04	1.45
C6-O6	1.45 ± 0.04	1.44 ± 0.04	1.45 ± 0.04	1.44 ± 0.04	1.45 ± 0.04	1.44 ± 0.04	1.42
O5-C1	1.45 ± 0.04	1.45 ± 0.04	1.45 ± 0.04	1.45 ± 0.04	1.45 ± 0.04	1.45 ± 0.04	1.43

Table 3: Bond lengths reported in Å as averages over 145 ns of MD in the NPT ensemble (after 5 ns equilibration). The error is the standard deviation.

	α-Glc		α-GlcN		α-GlcNAc		Exp
Bond	mutual pol.	no pol.	mutual pol.	no pol.	mutual pol.	no pol	
C1-C2	1.56 ± 0.04	1.57 ± 0.04	1.53 ± 0.04	1.53 ± 0.04	1.55 ± 0.04	1.56 ± 0.04	1.52

C2-C3	1.52 ± 0.04	1.53 ± 0.04	1.54 ± 0.04	1.54 ± 0.04	1.54 ± 0.04	1.54 ± 0.04	1.53
C3-C4	1.56 ± 0.04	1.56 ± 0.04	1.56 ± 0.04	1.56 ± 0.04	1.56 ± 0.04	1.56 ± 0.04	1.52
C4-C5	1.58 ± 0.04	1.58 ± 0.04	1.58 ± 0.04	1.58 ± 0.04	1.57 ± 0.04	1.57 ± 0.04	1.53
C5-C6	1.54 ± 0.04	1.55 ± 0.04	1.55 ± 0.04	1.55 ± 0.04	1.55 ± 0.04	1.55 ± 0.04	1.51
C1-O1	1.41 ± 0.03	1.41 ± 0.03	1.41 ± 0.03	1.41 ± 0.03	1.43 ± 0.03	1.43 ± 0.03	1.40
C2-O2	1.44 ± 0.04	1.45 ± 0.04	1.49 ± 0.03	1.49 ± 0.03	1.48 ± 0.03	1.48 ± 0.03	1.43
C3-O3	1.45 ± 0.04	1.44 ± 0.04	1.44 ± 0.04	1.44 ± 0.04	1.45 ± 0.04	1.45 ± 0.04	1.43
C4-O4	1.45 ± 0.04	1.45 ± 0.04	1.45 ± 0.04	1.45 ± 0.04	1.46 ± 0.04	1.45 ± 0.04	1.43
C5-O5	1.48 ± 0.04	1.48 ± 0.04	1.45 ± 0.04	1.45 ± 0.04	1.45 ± 0.04	1.44 ± 0.04	1.45
C6-O6	1.44 ± 0.04	1.44 ± 0.04	1.44 ± 0.04	1.44 ± 0.04	1.45 ± 0.04	1.44 ± 0.04	1.42
O5-C1	1.45 ± 0.04	1.45 ± 0.04	1.46 ± 0.04	1.45 ± 0.04	1.45 ± 0.04	1.45 ± 0.04	1.43

Table 4: Bond lengths reported in Å as averages over 145 ns of MD in the NPT ensemble (after 5 ns equilibration). The error is the standard deviation.

	α-Gal		α-GalN		α-GalNAc		Exp
Bond	mutual pol.	no pol.	mutual pol.	no pol.	mutual pol.	no pol	
C1-C2	1.56 ± 0.04	1.56 ± 0.04	1.56 ± 0.04	1.56 ± 0.04	1.56 ± 0.04	1.57 ± 0.04	1.52
C2-C3	1.55 ± 0.04	1.55 ± 0.04	1.54 ± 0.04	1.54 ± 0.04	1.53 ± 0.04	1.54 ± 0.04	1.53
C3-C4	1.56 ± 0.04	1.56 ± 0.04	1.52 ± 0.04	1.53 ± 0.04	1.56 ± 0.04	1.57 ± 0.04	1.52
C4-C5	1.55 ± 0.04	1.55 ± 0.04	1.57 ± 0.04	1.58 ± 0.04	1.55 ± 0.04	1.55 ± 0.04	1.53
C5-C6	1.54 ± 0.04	1.55 ± 0.04	1.55 ± 0.04	1.55 ± 0.04	1.55 ± 0.04	1.55 ± 0.04	1.51
C1-O1	1.43 ± 0.03	1.43 ± 0.03	1.41 ± 0.03	1.41 ± 0.03	1.43 ± 0.03	1.43 ± 0.03	1.40
C2-O2	1.45 ± 0.04	1.45 ± 0.04	1.49 ± 0.04	1.49 ± 0.04	1.47 ± 0.04	1.48 ± 0.03	1.43
C3-O3	1.46 ± 0.04	1.45 ± 0.04	1.46 ± 0.04	1.46 ± 0.04	1.46 ± 0.04	1.46 ± 0.04	1.43
C4-O4	1.46 ± 0.04	1.45 ± 0.04	1.47 ± 0.04	1.46 ± 0.04	1.45 ± 0.04	1.45 ± 0.04	1.43
C5-O5	1.44 ± 0.04	1.44 ± 0.04	1.45 ± 0.04	1.45 ± 0.04	1.48 ± 0.04	1.48 ± 0.04	1.45
C6-O6	1.45 ± 0.04	1.44 ± 0.04	1.45 ± 0.04	1.45 ± 0.04	1.45 ± 0.04	1.44 ± 0.04	1.42

O5-C1	1.46 ± 0.04	1.45 ± 0.04	1.48 ± 0.04	1.48 ± 0.04	1.44 ± 0.04	1.45 ± 0.04	1.43

Table 5: Angle values reported in ° as averages over 145 ns of MD in the NPT ensemble (after 5 ns equilibration). The error is the standard deviation.

	β-Glc		β-GlcN		β-GlcNAc		Exp
Angle	mutual pol.	no pol.	mutual pol.	no pol.	mutual pol.	no pol	
C1-C2-C3	109 ± 3	109 ± 3	108 ± 2	109± 3	109 ± 3	110 ± 3	110
C2-C3-C4	110 ± 3	112 ± 3	111 ± 3	112 ± 3	111 ± 3	111 ± 3	110
C3-C4-C5	110 ± 3	109 ± 3	109 ± 3	109 ± 3	110 ± 3	110 ± 3	110
C4-C5-C6	114 ± 3	115 ± 3	114 ± 3	114 ± 3	114 ± 3	114 ± 3	113
C4-C5-O5	109 ± 3	103 ± 3	109 ± 3	109 ± 3	109 ± 3	108 ± 3	109
C5-O5-C1	113 ± 3	109 ± 3	113 ± 3	113 ± 3	113 ± 3	114 ± 3	113
C5-C6-O6	108 ± 3	110 ± 3	109 ± 3	109 ± 3	114 ± 3	114 ± 3	111
O5-C1-C2	109 ± 3	104 ± 3	109 ± 3	110 ± 3	110 ± 3	110 ± 3	110
O5-C5-C6	107 ± 3	104 ± 3	107 ± 3	107 ± 3	107 ± 3	107 ± 3	107
O5-C1-O1	107 ± 3	108 ± 3	105 ± 3	106 ± 3	105 ± 3	105 ± 3	109
C2-C1-O1	108 ± 3	108± 3	109± 3	109± 3	109 ± 3	110 ± 3	110
C1-C2-O2	112 ± 3	112 ± 3	110 ± 2	110 ± 3	112 ± 3	112 ± 3	109
C3-C2-O2	107 ± 3	108 ± 3	110 ± 2	110 ± 3	111 ± 3	111 ± 3	111
C2-C3-O3	110 ± 3	112 ± 3	112 ± 3	112 ± 3	109 ± 3	109 ± 3	108
C4-C3-O3	107 ± 3	107 ± 3	107 ± 3	107 ± 3	111 ± 3	111 ± 3	111
C3-C4-O4	110 ± 3	109 ± 3	110 ± 3	110 ± 3	105 ± 3	105 ± 3	110
C5-C4-O4	110 ± 3	111 ± 3	110 ± 3	109 ± 3	113 ± 3	113 ± 3	110

Table 6: Angle values reported in ° as averages over 145 ns of MD in the NPT ensemble (after 5 ns equilibration). The error is the standard deviation.

	β-Gal		β-GalN		β-GalNAc		Exp

Angle	mutual pol.	no pol.	mutual pol.	no pol.	mutual pol.	no pol	
C1-C2-C3	110 ± 3	110 ± 3	109 ± 2	109± 2	109 ± 3	110 ± 3	110
C2-C3-C4	109 ± 3	110 ± 3	110 ± 2	111 ± 3	110 ± 3	110 ± 3	110
C3-C4-C5	109 ± 3	109 ± 3	110 ± 3	110 ± 3	110 ± 3	110 ± 3	110
C4-C5-C6	113 ± 3	113 ± 3	114 ± 3	114 ± 3	115 ± 3	115 ± 3	113
C4-C5-O5	110 ± 3	110 ± 3	110 ± 3	110 ± 3	109 ± 3	109 ± 3	109
C5-O5-C1	112 ± 3	113 ± 3	113 ± 3	113 ± 3	114 ± 3	114 ± 3	113
C5-C6-O6	108 ± 3	109 ± 3	109 ± 3	109 ± 3	111 ± 3	112 ± 3	111
O5-C1-C2	109 ± 3	110 ± 3	110 ± 3	110 ± 3	110 ± 3	110 ± 3	110
O5-C5-C6	108 ± 3	108 ± 3	108 ± 3	108 ± 3	106 ± 3	107 ± 3	107
O5-C1-O1	107 ± 3	107 ± 3	106 ± 3	106 ± 3	105 ± 3	105 ± 3	109
C2-C1-O1	108 ± 3	108± 3	109± 3	109± 3	109 ± 3	109 ± 3	110
C1-C2-O2	112 ± 3	112 ± 3	116 ± 3	116 ± 3	111 ± 3	112 ± 3	109
C3-C2-O2	107 ± 3	107 ± 3	109 ± 2	109 ± 2	112 ± 3	113 ± 3	111
C2-C3-O3	112 ± 3	112 ± 3	112 ± 3	112 ± 3	109 ± 3	109 ± 3	108
C4-C3-O3	108 ± 3	108 ± 3	108 ± 3	108 ± 3	110 ± 3	110 ± 3	111
C3-C4-O4	110 ± 3	110 ± 3	110 ± 3	110 ± 3	110 ± 3	110 ± 3	110
C5-C4-O4	109 ± 3	109 ± 3	108 ± 3	108 ± 3	109 ± 3	109 ± 3	110

Table 7: Angle values reported in ° as averages over 145 ns of MD in the NPT ensemble (after 5 ns equilibration). The error is the standard deviation.

	α-Glc		α-GlcN		α-GlcNAc		Exp
Angle	mutual pol.	no pol.	mutual pol.	no pol.	mutual pol.	no pol	
C1-C2-C3	111 ± 3	111 ± 3	110 ± 2	110 ± 2	110 ± 3	111 ± 3	110
C2-C3-C4	110 ± 3	110 ± 3	111 ± 3	112 ± 2	111 ± 2	111 ± 3	110
C3-C4-C5	109 ± 3	109 ± 3	109 ± 3	110 ± 3	112 ± 2	110 ± 3	110

C4-C5-C6	112 ± 3	112 ± 3	113 ± 3	114 ± 3	113 ± 3	112 ± 3	113
C4-C5-O5	113 ± 3	113 ± 3	112 ± 3	112 ± 3	112 ± 3	110 ± 3	109
C5-O5-C1	115 ± 3	115 ± 3	114 ± 3	114 ± 3	117 ± 3	115 ± 3	113
C5-C6-O6	109 ± 3	109 ± 3	108 ± 3	108 ± 3	108 ± 3	108 ± 3	111
O5-C1-C2	112 ± 3	112 ± 3	110 ± 3	110 ± 3	110 ± 3	110 ± 3	110
O5-C5-C6	111 ± 3	111 ± 3	113 ± 3	114 ± 3	110 ± 3	107 ± 3	107
O5-C1-O1	110 ± 3	109 ± 3	107 ± 4	107 ± 3	110 ± 3	113 ± 3	109
C2-C1-O1	109 ± 3	109 ± 3	110 ± 3	110 ± 3	107 ± 3	108 ± 3	110
C1-C2-O2	111 ± 3	111 ± 3	116 ± 3	116 ± 2	114 ± 3	113 ± 3	109
C3-C2-O2	108 ± 3	108 ± 3	108 ± 2	108 ± 2	110 ± 3	110 ± 3	111
C2-C3-O3	112 ± 3	112 ± 3	111 ± 3	111 ± 3	111 ± 3	111 ± 3	108
C4-C3-O3	109 ± 3	109 ± 3	111 ± 3	111 ± 3	110 ± 3	109 ± 3	111
C3-C4-O4	114 ± 3	114 ± 3	114 ± 3	114 ± 3	112 ± 3	111 ± 3	110
C5-C4-O4	111 ± 3	111 ± 3	110 ± 3	110 ± 3	110 ± 3	112 ± 3	110

Table 8: Angle values reported in ° as averages over 145 ns of MD in the NPT ensemble (after 5 ns equilibration). The error is the standard deviation.

	α-Gal		α-GalN		α-GalNAc		Exp
Angle	mutual pol.	no pol.	mutual pol.	no pol.	mutual pol.	no pol	
C1-C2-C3	110 ± 3	110 ± 3	111 ± 2	111 ± 2	110 ± 3	111 ± 3	110
C2-C3-C4	109 ± 3	110 ± 3	110 ± 3	110 ± 3	110 ± 3	110 ± 3	110
C3-C4-C5	109 ± 3	109 ± 3	110 ± 3	109 ± 3	110 ± 3	110 ± 3	110
C4-C5-C6	113 ± 3	113 ± 3	112 ± 3	112 ± 3	115 ± 3	115 ± 3	113
C4-C5-O5	111 ± 3	111 ± 3	112 ± 3	111 ± 3	110 ± 3	110 ± 3	109
C5-O5-C1	114 ± 3	114 ± 3	113 ± 3	113 ± 3	115 ± 3	115 ± 3	113
C5-C6-O6	108 ± 3	109 ± 3	107 ± 3	108 ± 3	111 ± 3	111 ± 3	111
O5-C1-C2	110 ± 3	110 ± 3	112 ± 3	112 ± 3	111 ± 3	111 ± 3	110

O5-C5-C6	108 ± 3	108 ± 3	110 ± 3	110 ± 3	105 ± 3	106 ± 3	107
O5-C1-O1	113 ± 3	113 ± 3	109 ± 3	108 ± 3	112 ± 3	112 ± 3	109
C2-C1-O1	109 ± 3	109 ± 3	112 ± 3	112 ± 3	109 ± 3	109 ± 3	110
C1-C2-O2	114 ± 3	114 ± 3	111 ± 2	111 ± 2	113 ± 3	113 ± 3	109
C3-C2-O2	107 ± 3	107 ± 3	109 ± 2	109 ± 2	110 ± 3	110 ± 3	111
C2-C3-O3	111 ± 3	111 ± 3	111 ± 3	112 ± 3	110 ± 3	110 ± 3	108
C4-C3-O3	107 ± 3	107 ± 3	110 ± 3	107 ± 3	108 ± 3	108 ± 3	111
C3-C4-O4	109 ± 3	110 ± 3	107 ± 3	111 ± 3	110 ± 3	110 ± 3	110
C5-C4-O4	109 ± 3	109 ± 3	114 ± 3	114 ± 3	110 ± 3	109 ± 3	110

Table 9: Torsion values reported in ° as averages over 145 ns of MD in the NPT ensemble (after 5 ns equilibration). The error is the standard deviation.

	β-Glc		β-GlcN		β-GlcNAc		Exp
Torsion	mutual pol.	no pol.	mutual pol.	no pol.	mutual pol.	no pol	
C1-C2-C3-C4	-55± 6	-50± 6	-55 ± 5	-55± 6	-54± 6	-52± 6	-54
C2-C3-C4-C5	53 ± 6	50 ± 6	54 ± 5	53 ± 6	54 ± 6	53 ± 6	55
C3-C4-C5-O5	-55 ± 6	-58± 6	-55± 4	-55± 6	-55± 6	-56± 6	-57
C4-C5-O5-C1	62 ± 5	75 ± 6	62 ± 5	62 ± 6	61 ± 6	62 ± 6	62
C5-O5-C1-C2	-65 ± 6	-77± 6	-65 ± 5	-64± 6	-63± 5	-62± 6	-62
O5-C1-C2-C3	59 ± 6	61 ± 6	58 ± 5	57 ± 6	57 ± 6	55 ± 6	57
O1-C1-O5-C5	178± 6	168± 6	178± 6	178± 7	180± 7	180± 7	N/A
O2-C2-C1-O5	178± 6	180± 6	179± 5	178± 7	180± 7	180± 7	N/A
O3-C3-C2-C1	-174± 6	-170± 6	-175± 6	-174± 6	-177± 6	-175± 6	N/A
O4-C4-C3-C2	174± 6	170± 6	174± 6	174± 6	176± 6	176± 6	N/A

Table 10: Torsion values reported in ° as averages over 145 ns of MD in the NPT ensemble (after 5 ns equilibration). The error is the standard deviation.

	β-Gal		β-GalN		β-GalNAc		Exp
Torsion	mutual pol.	no pol.	mutual pol.	no pol.	mutual pol.	no pol	
C1-C2-C3-C4	-55 ± 5	-54 ± 5	-53 ± 5	-52± 5	-53± 5	-52± 6	-54
C2-C3-C4-C5	53 ± 5	53 ± 5	54 ± 5	53 ± 5	54 ± 5	54± 5	55
C3-C4-C5-O5	-57 ± 5	-57± 5	-57± 5	-57± 5	-57 ± 5	-57± 5	-57
C4-C5-O5-C1	63 ± 6	63 ± 6	63 ± 6	63± 6	62± 6	62± 5	62
C5-O5-C1-C2	-64 ± 6	-63 ± 6	-62 ± 6	-61 ± 6	-62 ± 5	-61± 6	-62
O5-C1-C2-C3	59 ± 6	57 ± 6	56 ± 6	55 ± 6	56 ± 6	54± 6	57
O1-C1-O5-C5	179 ± 6	180 ± 6	180 ± 6	-179 ± 6	-179 ± 6	-179± 6	N/A
O2-C2-C1-O5	178 ± 6	176 ± 6	180 ± 6	178 ± 6	180 ± 6	-179± 6	N/A
O3-C3-C2-C1	-175 ± 6	-174 ± 6	-173 ± 6	-172 ± 6	-174 ± 6	-173± 6	N/A
O4-C4-C3-C2	-66 ± 6	-66 ± 6	-66 ± 6	-66 ± 6	-66 ± 6	-66± 6	N/A

Table 11: Torsion values reported in ° as averages over 145 ns of MD in the NPT ensemble (after 5 ns equilibration). The error is the standard deviation.

	α-Glc		α-GlcN		α-GlcNAc		Exp
Torsion	mutual pol.	no pol.	mutual pol.	no pol.	mutual pol.	no pol	
C1-C2-C3-C4	-57 ± 9	-52 ±13	13 ± 52	52 ± 13	55 ± 5	-52± 6	-54
C2-C3-C4-C5	56 ± 8	57 ± 7	-8 ± 52	-46 ± 17	-49 ± 5	52± 6	55
C3-C4-C5-O5	-49 ±15	-48 ±14	11 ± 48	47 ± 13	45 ± 6	-54± 6	-57
C4-C5-O5-C1	43 ±29	35 ±36	-21 ± 51	-57 ± 7	-51 ± 6	59± 7	62
C5-O5-C1-C2	-42 ±33	-28 ±47	27 ± 54	61 ± 6	58 ± 6	-59± 6	-62
O5-C1-C2-C3	49 ±22	37 ±34	-22 ± 51	-58 ± 7	-58 ± 5	54± 6	57
O1-C1-O5-C5	80 ±33	94 ±46	147 ± 51	-179 ± 6	176 ± 6	62± 7	N/A
O2-C2-C1-O5	170±22	158 ±35	102 ± 51	66 ± 7	66 ± 6	178± 6	N/A
O3-C3-C2-C1	-178±9	-173 ±13	-111 ± 51	-73 ± 13	-68 ± 6	-174± 6	N/A

O4-C4-C3-C2	-179± 8	-179 ± 7	116 ± 53	77 ± 17	75 ± 6	176± 7	N/A

Table 12: Torsion values reported in ° as averages over 145 ns of MD in the NPT ensemble (after 5 ns equilibration). The error is the standard deviation.

	α-Gal		α-GalN		α-GalNAc		Exp
Torsion	mutual pol.	no pol.	mutual pol.	no pol.	mutual pol.	no pol	
C1-C2-C3-C4	-55 ± 5	-54 ± 5	-52 ±10	-54 ± 5	-55 ± 5	-54± 5	-54
C2-C3-C4-C5	55 ± 5	54 ± 5	55 ± 6	55 ± 5	55 ± 5	55± 5	55
C3-C4-C5-O5	-56 ± 6	-56 ± 5	-56 ± 8	-57 ± 6	-55 ± 6	-56± 5	-57
C4-C5-O5-C1	59 ± 6	60 ± 6	54 ±15	57 ± 6	57 ± 6	58± 6	62
C5-O5-C1-C2	-57 ± 7	-58 ± 6	-50 ±20	-55 ± 6	-56 ± 6	-57± 6	-62
O5-C1-C2-C3	55 ± 6	54 ± 6	49 ±18	53 ± 6	55 ± 6	54± 6	57
O1-C1-O5-C5	65 ± 8	64 ± 7	71 ± 9	68 ± 7	65 ± 7	65± 7	N/A
O2-C2-C1-O5	175± 7	175 ± 6	172 ± 6	174 ± 6	178 ± 6	177± 6	N/A
O3-C3-C2-C1	-173± 6	-172 ± 6	-173 ± 6	-177 ± 6	-174 ± 6	-173± 6	N/A
O4-C4-C3-C2	-65± 6	-65 ± 6	-66 ± 6	-69 ± 6	-65 ± 6	-65± 6	N/A

Radial distribution function for α anomers

Table 13: First peak position (r_0), first minimum position ($r_{\min}$) and coordination number (CN) for hydroxyl-water radial distribution function of $\alpha-$Glc with and without mutual polarization.

	Mutual pol.			No pol.		
Oxygen	r_0 (Å)	$r_{\min}$ (Å)	CN	r_0 (Å)	$r_{\min}$ (Å)	CN
O_1	2.85	3.65	4.07	3.05	3.95	4.41
O_2	2.85	3.35	3.33	3.05	4.05	4.84
O_3	2.85	3.65	4.42	3.05	3.95	4.63
O_4	2.85	3.45	3.39	3.05	3.85	3.93
Average	2.85	3.45	3.51	3.05	4.05	4.82
O_6	2.85	3.35	3.33	3.05	3.95	4.73

Mean square displacement of $\beta-$Glc center-of-mass as a function of concentration

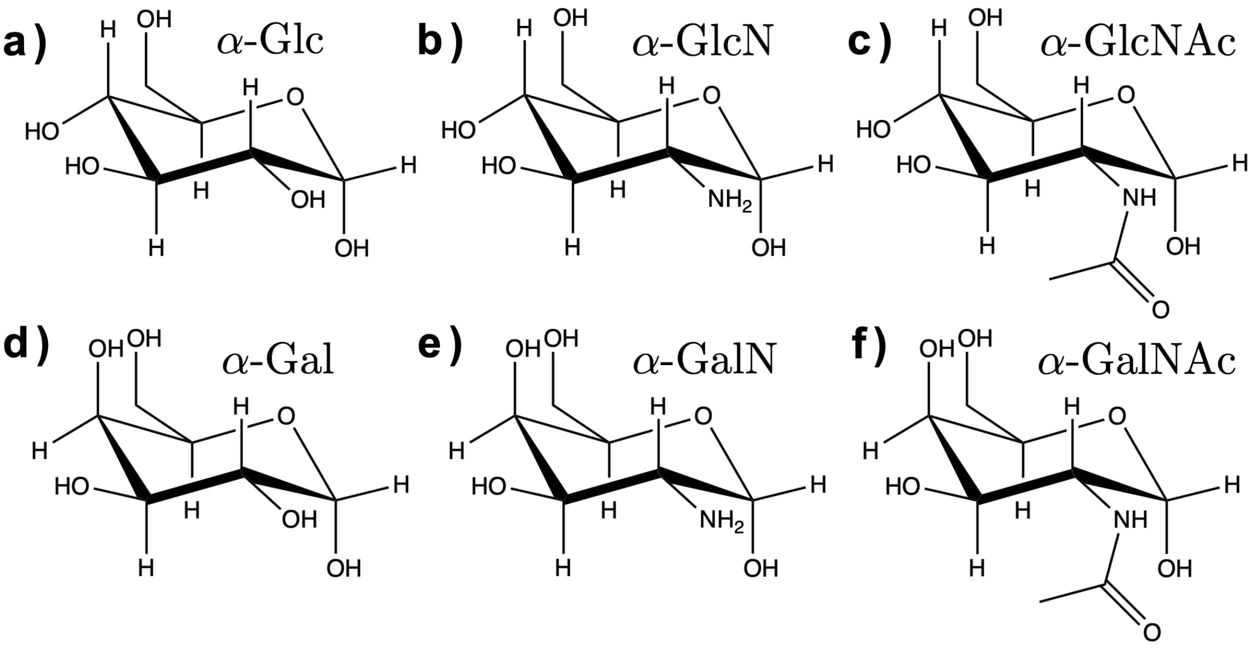

Figure S1: Chemical structure of the α anomers of the six monosaccharides studied here. a) Glc, b) GlcN, c) GlcNAc, d) Gal, e) GalN, f) GalNAc.

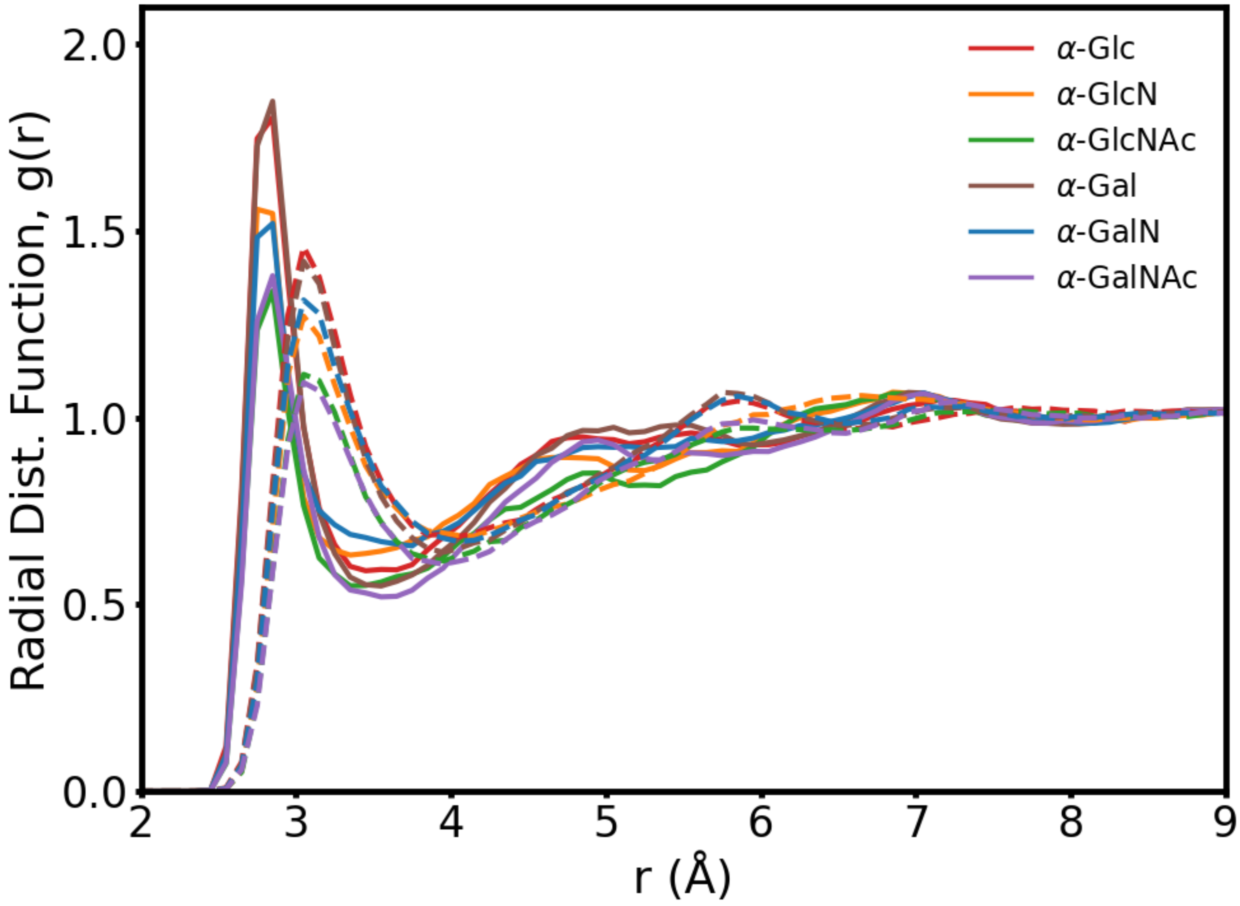

Figure S2: Carbohydrate-water radial distribution function for the six α anomers, with (solid lines) and without (dotted lines) mutual polarization. Each curve is an average of the $O_1 - O_w$, $O_2/N_2 - O_w$, $O_3 - O_w$ and $O_4 - O_w$ distribution functions, where O_w is the water oxygen.

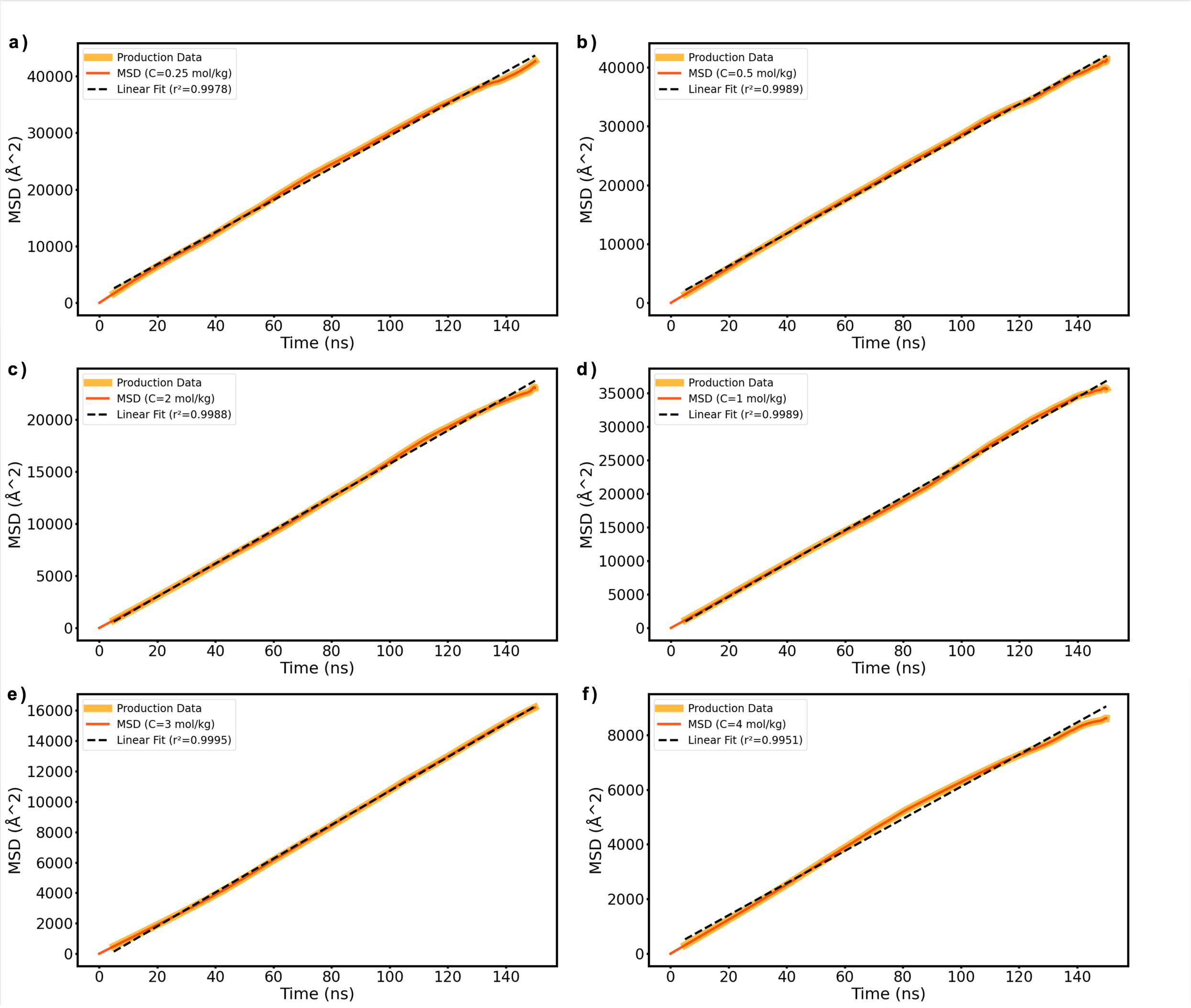

Figure S3: Mean square displacement of $\beta-$Glc center-of-mass as a function of MD time and linear fit a) c=0.25 mol/kg (29 $\beta-$Glc molecules), b) c=0.5 mol/kg (56 $\beta-$Glc molecules), c) c=1 mol/kg (105 $\beta-$Glc molecules), d) c=2 mol/kg (188 $\beta-$Glc molecules), e) c=3 mol/kg (255 $\beta-$Glc molecules), f) c=4 mol/kg (305 $\beta-$Glc molecules).

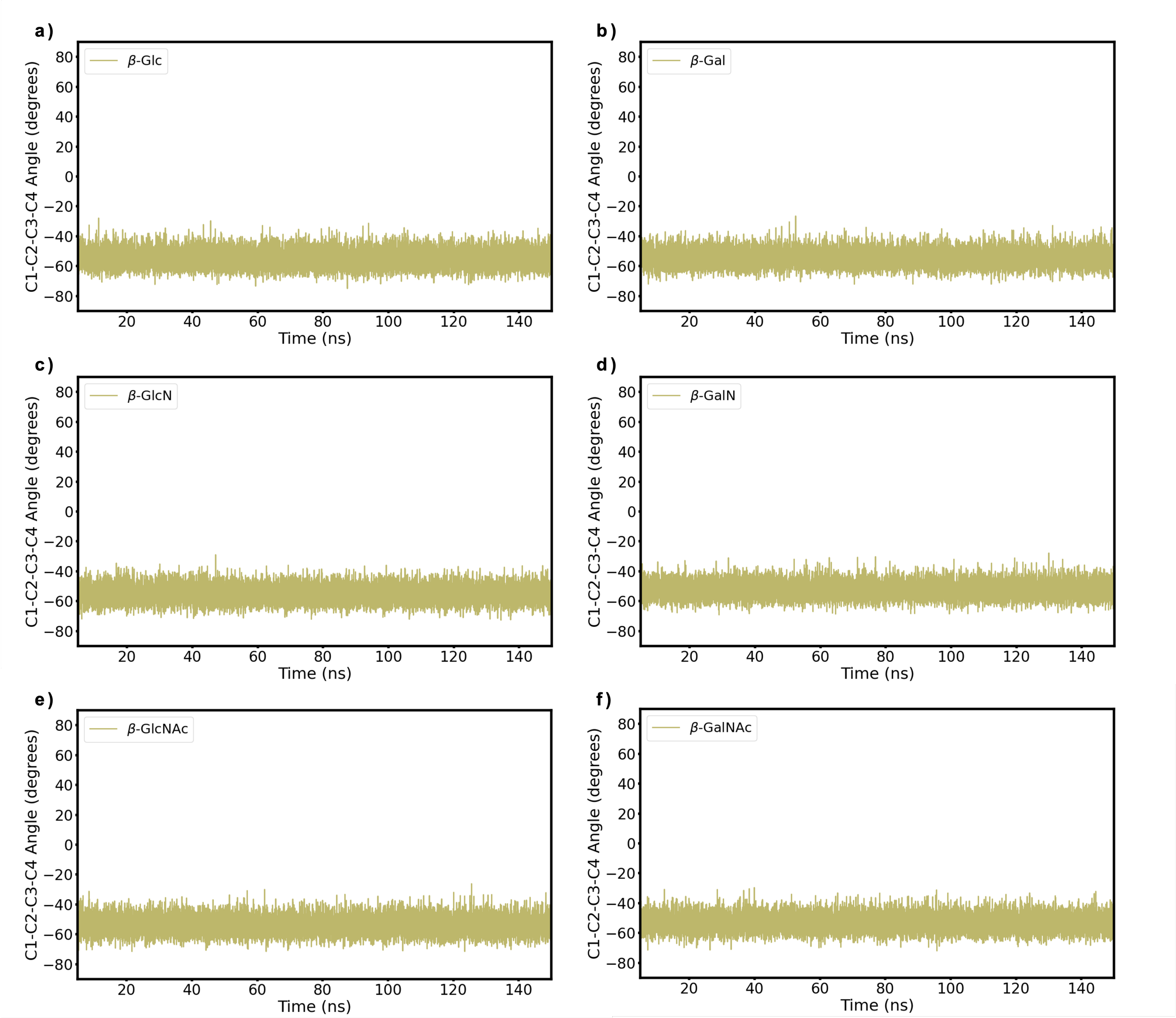

Figure S4: Time evolution of the torsion angle $C_1 - C_2 - C_3 - C_4$ in $\beta-$ anomers during 145 ns production MD with AMOEBA (mutual polarization, β parameters). $C_1 - C_2 - C_3 - C_4$ is -55° when in chair conformation. a) β-Glc, b) β-GlcN, c) β-GlcNAc, d) β-Gal, e) β-GalN, f) β-GalNAc.

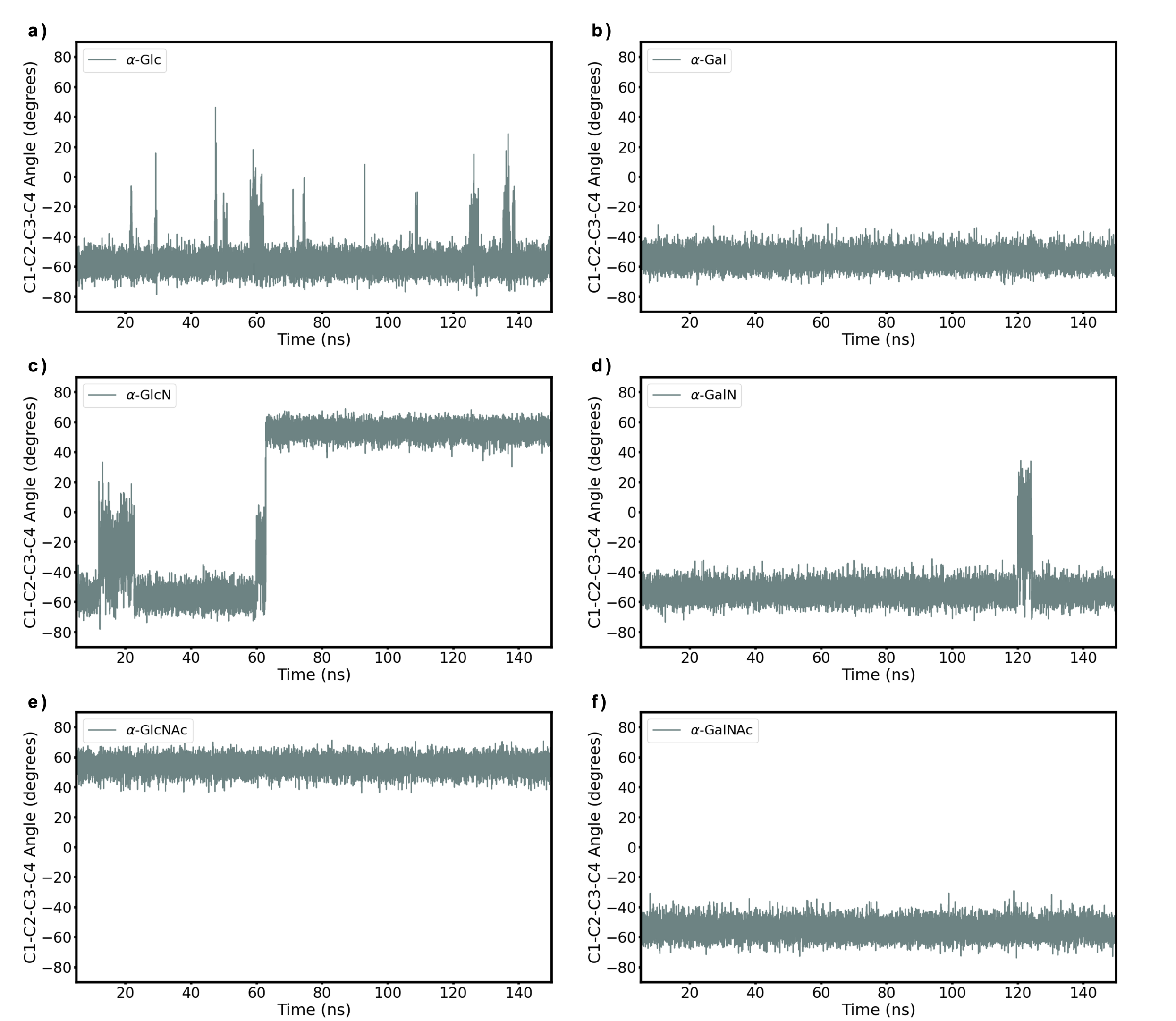

Figure S5: Time evolution of the torsion angle $C_1 - C_2 - C_3 - C_4$ in $\alpha-$ anomers during 145 ns production MD with AMOEBA (mutual polarization, α parameters). $C_1 - C_2 - C_3 - C_4$ is -55° when in chair conformation. a) α-Glc, b) α-GlcN, c) α-GlcNAc, d) α-Gal, e) α-GalN, f) α-GalNAc. Overall, α-Glc, α-GlcN, α-GlcNAc, α-Gal, α-GalN and α-GalNAc spends 2.9, 7.4, 0.09, 0.07, 2.6 and 0.05% of the time, respectively, in a conformation other than chair.

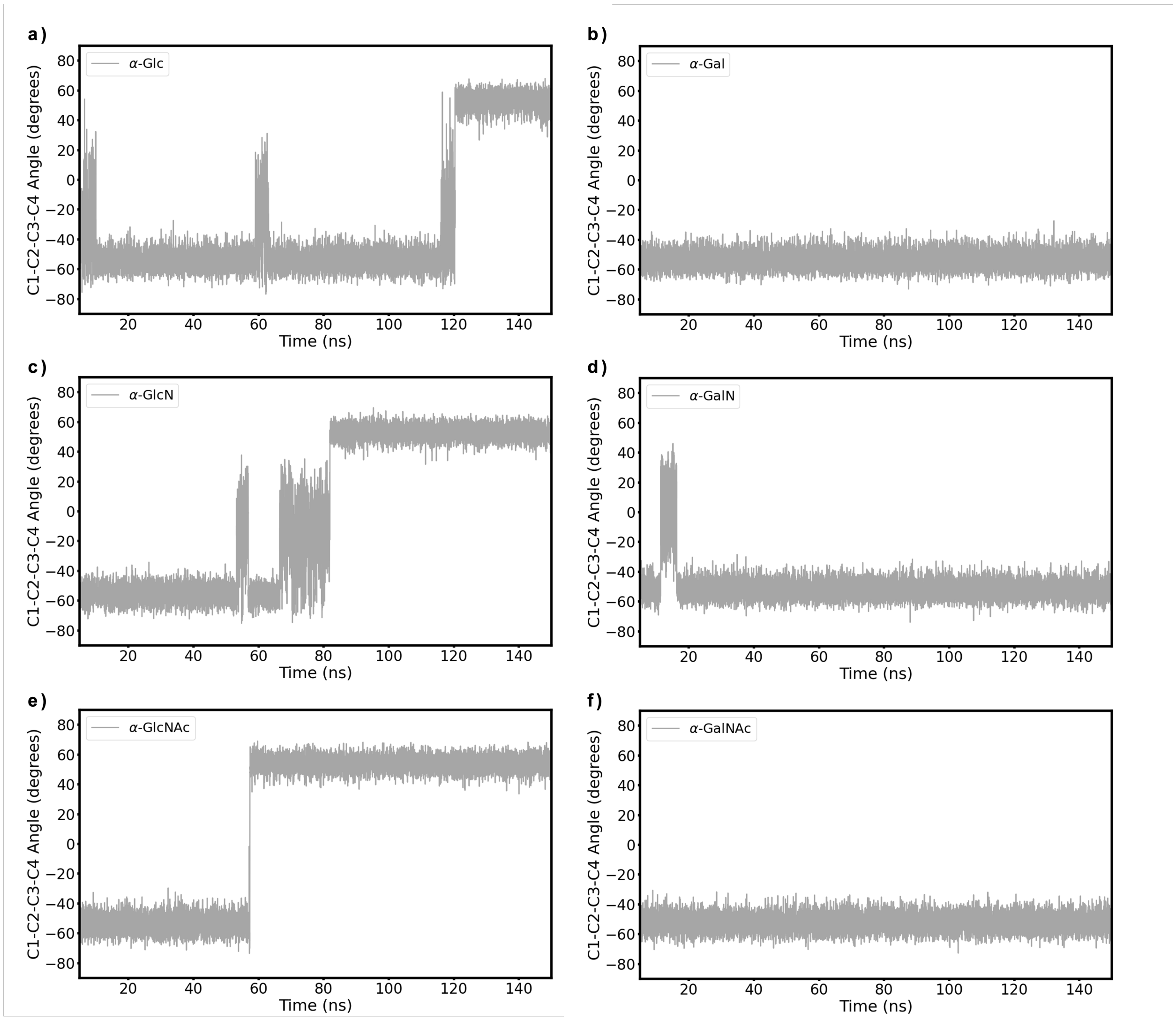

Figure S6: Time evolution of the torsion angle $C_1 - C_2 - C_3 - C_4$ in $\alpha-$ anomers during 145 ns production MD with AMOEBA (mutual polarization, β parameters). $C_1 - C_2 - C_3 - C_4$ is -55° when in chair conformation. a) α-Glc, b) α-GlcN, c) α-GlcNAc, d) α-Gal, e) α-GalN, f) α-GalNAc. Overall, α-Glc, α-GlcN, α-GlcNAc, α-Gal, α-GalN and α-GalNAc spends 7.6, 10.9, 0.1, 0.1, 4.1 and 0.2% of the time, respectively, in a conformation other than chair.

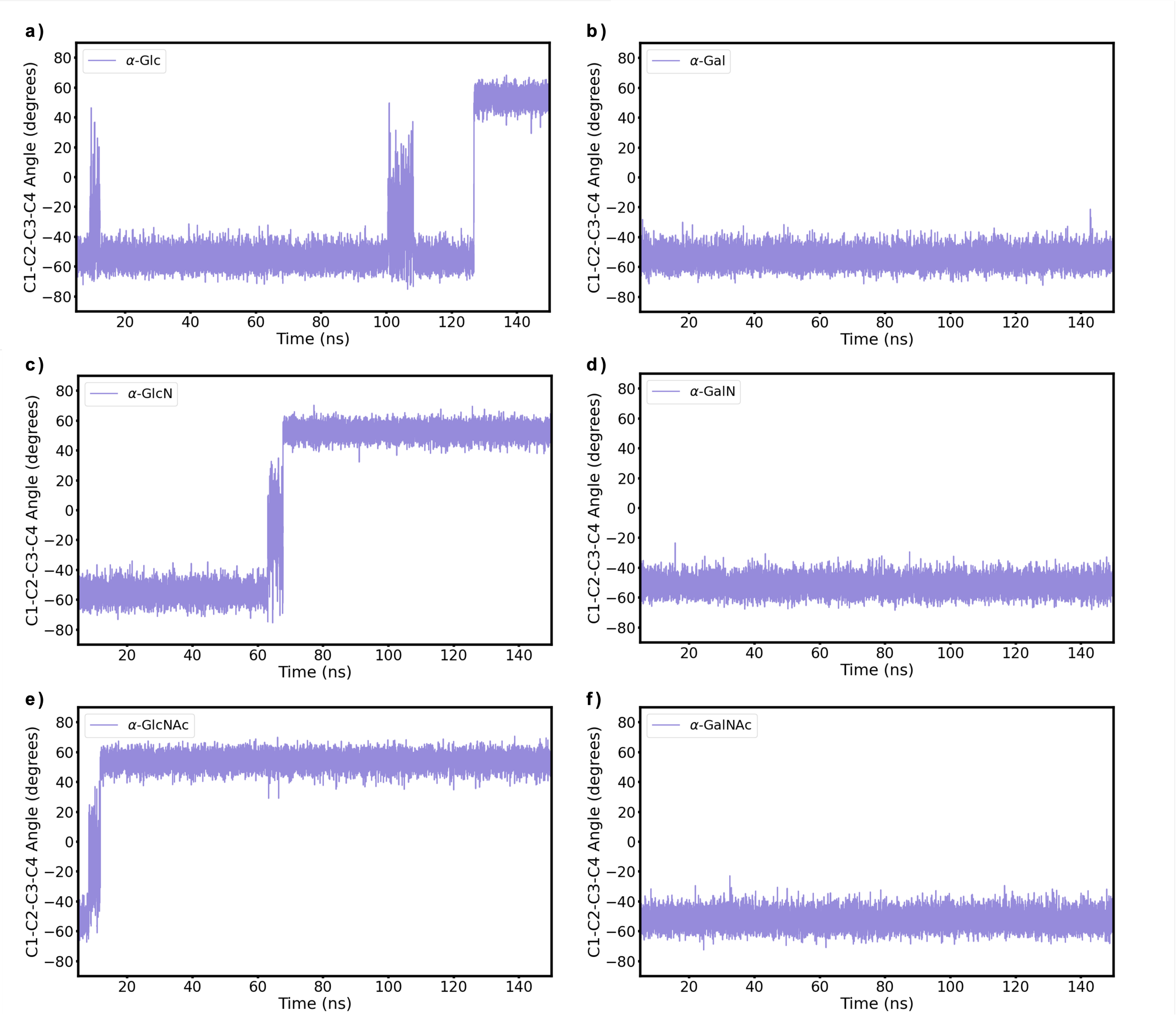

Figure S7: Time evolution of the torsion angle $C_1-C_2-C_3-C_4$ in $\alpha-$ anomers during 145 ns production MD with AMOEBA (mutual polarization, α parameters, no energy minimization prior to MD). $C_1-C_2-C_3-C_4$ is -55° when in chair conformation. a) α-Glc, b) α-GlcN, c) α-GlcNAc, d) α-Gal, e) α-GalN, f) α-GalNAc. Overall, α-Glc, α-GlcN, α-GlcNAc, α-Gal, α-GalN and α-GalNAc spends 4.9, 2.7, 2.4, 0.1, 0.2 and 0.2% of the time, respectively, in a conformation other than chair.

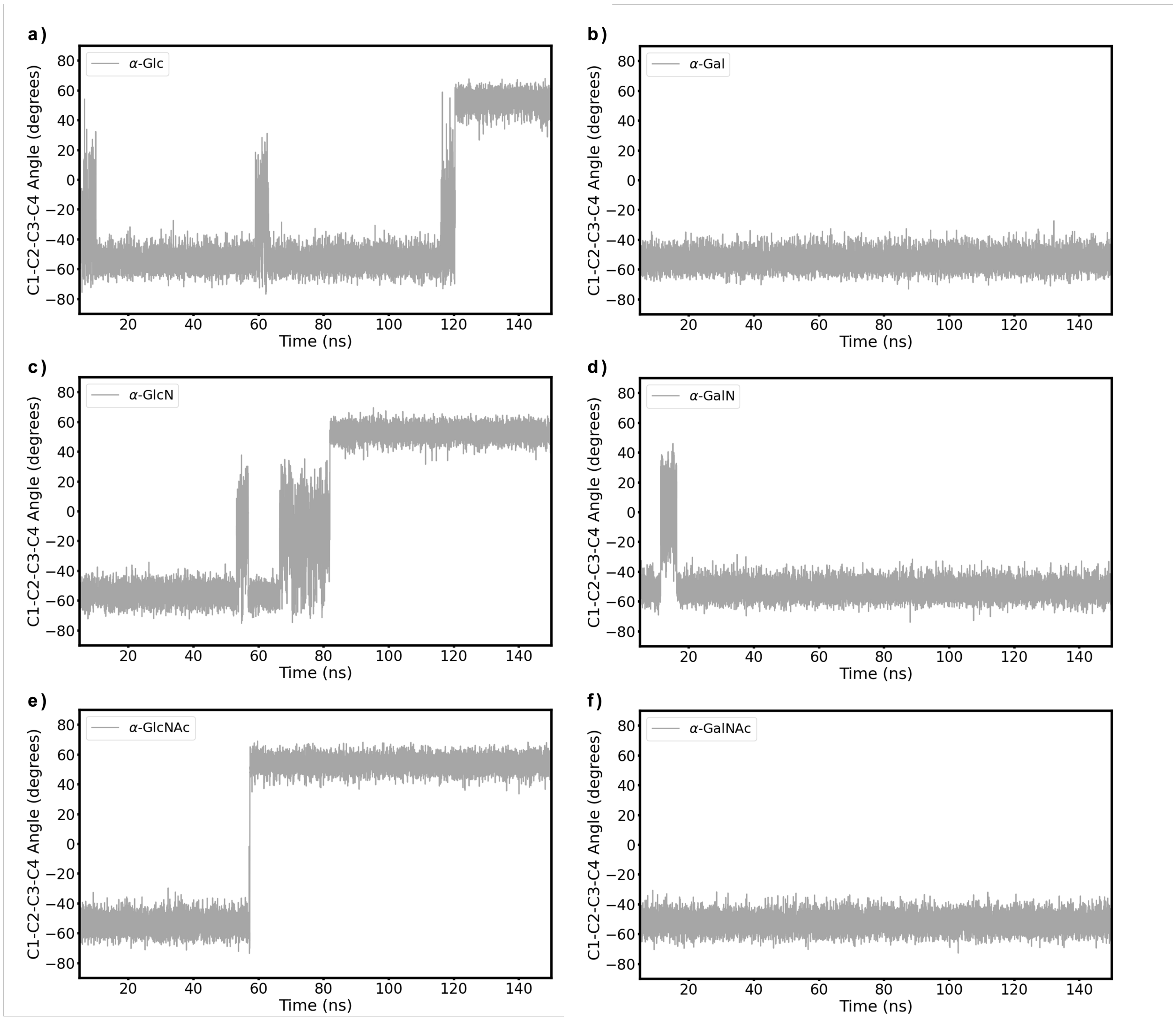

Figure S8: Time evolution of the torsion angle $C_1 - C_2 - C_3 - C_4$ in $\alpha-$ anomers during 145 ns production MD with AMOEBA (mutual polarization, β parameters). $C_1 - C_2 - C_3 - C_4$ is -55° when in chair conformation. a) α-Glc, b) α-GlcN, c) α-GlcNAc, d) α-Gal, e) α-GalN, f) α-GalNAc. Overall, α-Glc, α-GlcN, α-GlcNAc, α-Gal, α-GalN and α-GalNAc spends 7.6, 10.9, 0.1, 0.1, 4.1 and 0.2% of the time, respectively, in a conformation other than chair.

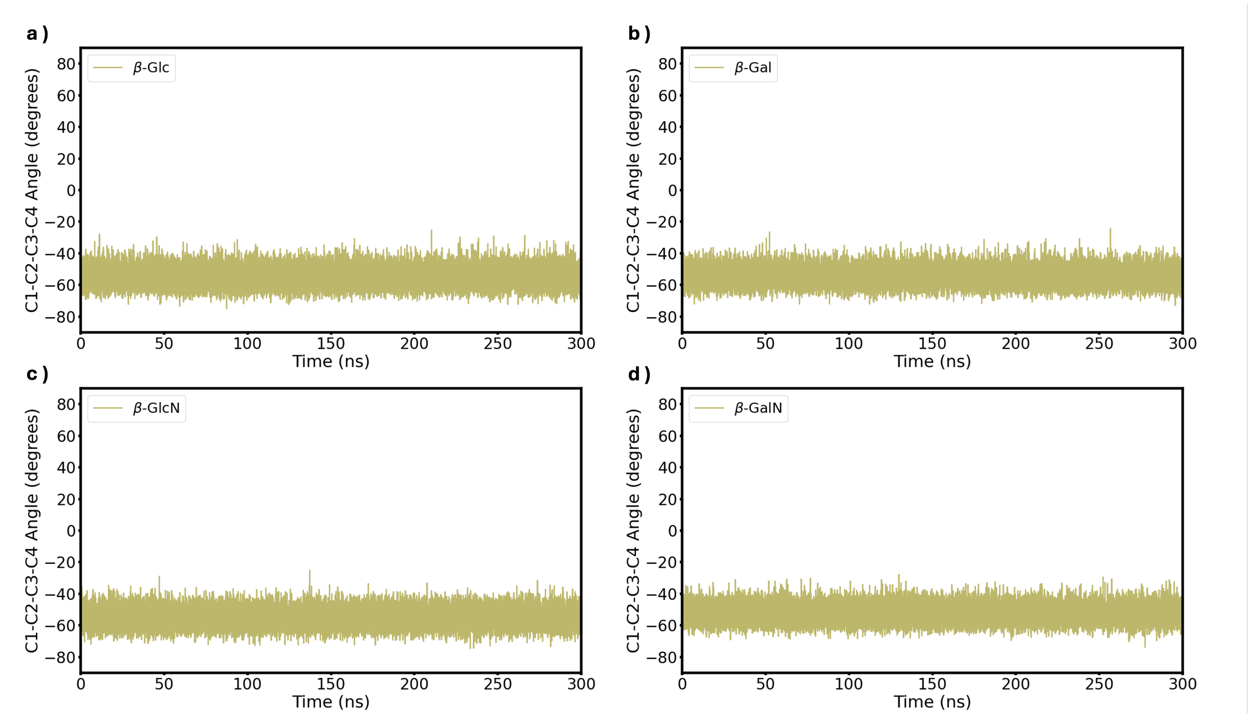

Figure S9: Time evolution of the torsion angle $C_1 - C_2 - C_3 - C_4$ in $\beta-$Glc, GlcN, Gal, and GalN during 300 ns (extension of the previous 145 ns presented in Figure S4), first replicate. $C_1 - C_2 - C_3 - C_4$ is -55° when in chair conformation. a) β-Glc, b) β-GlcN, c) β-GlcNAc, d) β-Gal.

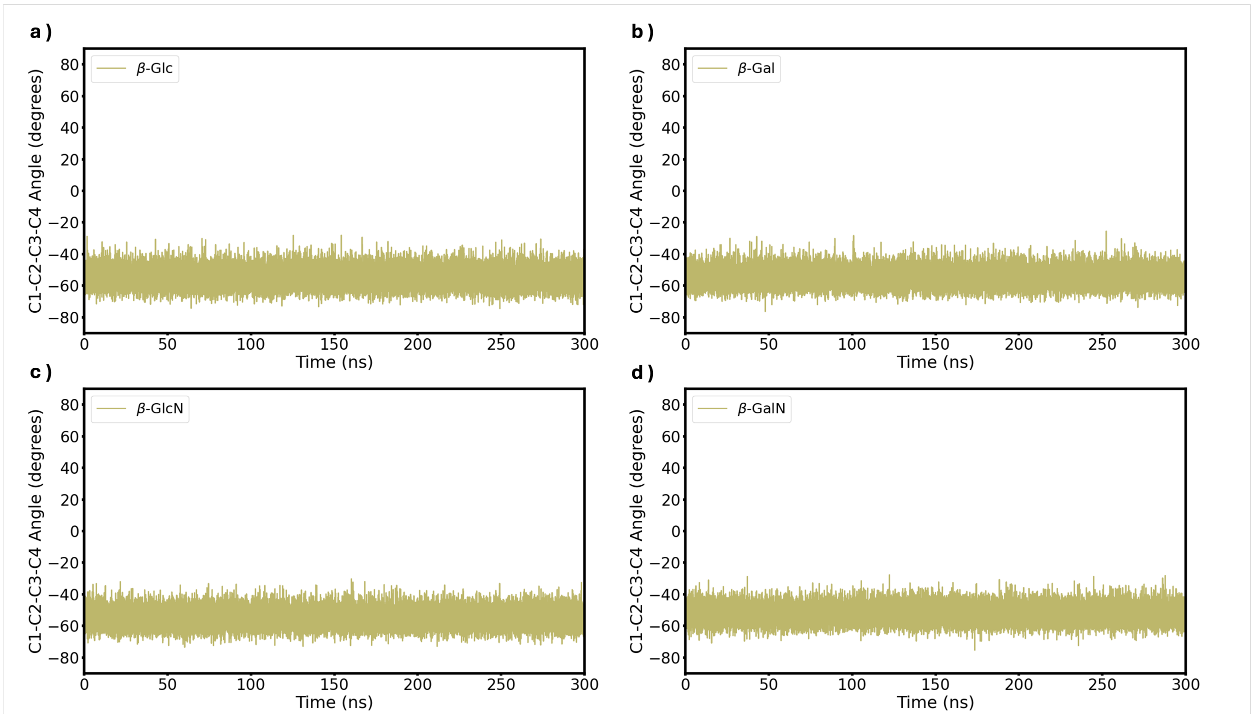

Figure S10: Time evolution of the torsion angle $C_1 - C_2 - C_3 - C_4$ in $\beta-$Glc, GlcN, Gal, and GalN during 300 ns, second replicate. $C_1 - C_2 - C_3 - C_4$ is -55° when in chair conformation. a) β-Glc, b) β-GlcN, c) β-GlcNAc, d) β-Gal.

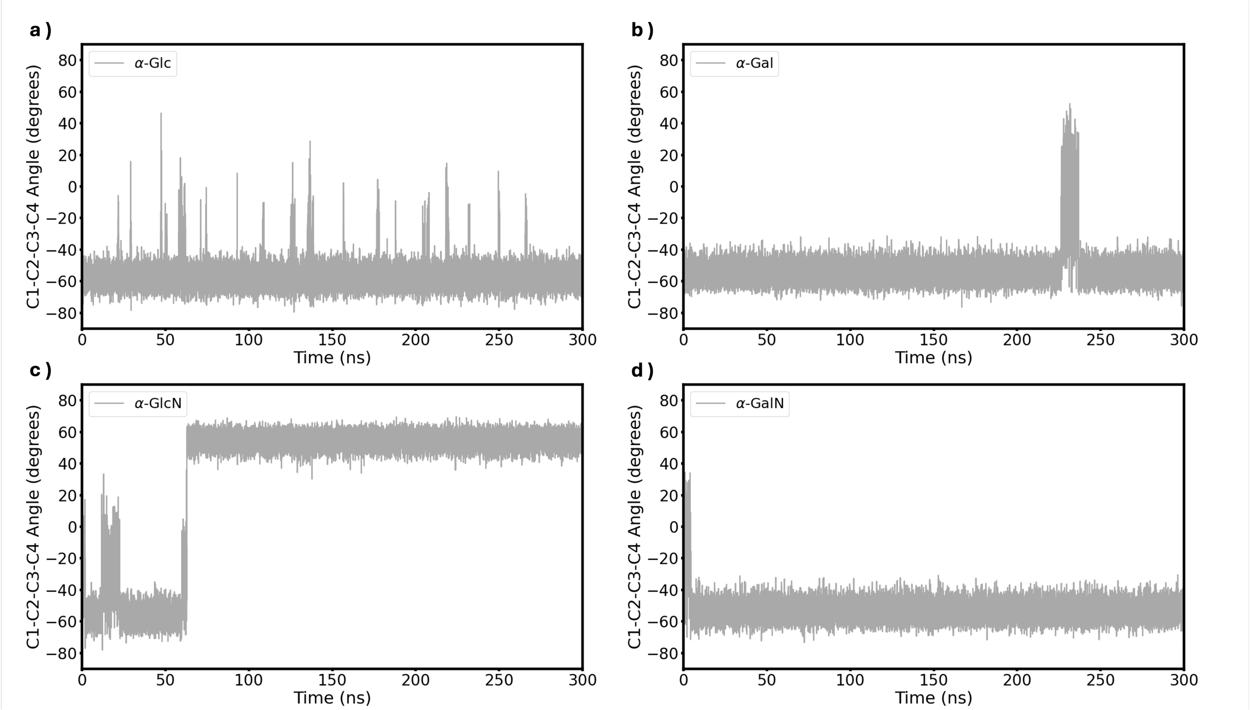

Figure S11: Time evolution of the torsion angle $C_1 - C_2 - C_3 - C_4$ in $\alpha-$Glc, GlcN, Gal, and GalN during 300 ns (extension of the previous 145 ns presented in Figure S5), first replicate. $C_1 - C_2 - C_3 - C_4$ is -55° when in chair conformation. a) α-Glc, b) α-GlcN, c) α-GlcNAc, d) α-Gal. Overall, α-Glc, α-GlcN, α-Gal, and α-GalN spends 2.3, 3.7, 3.1, 1.3% of the time, respectively, in a conformation other than chair.

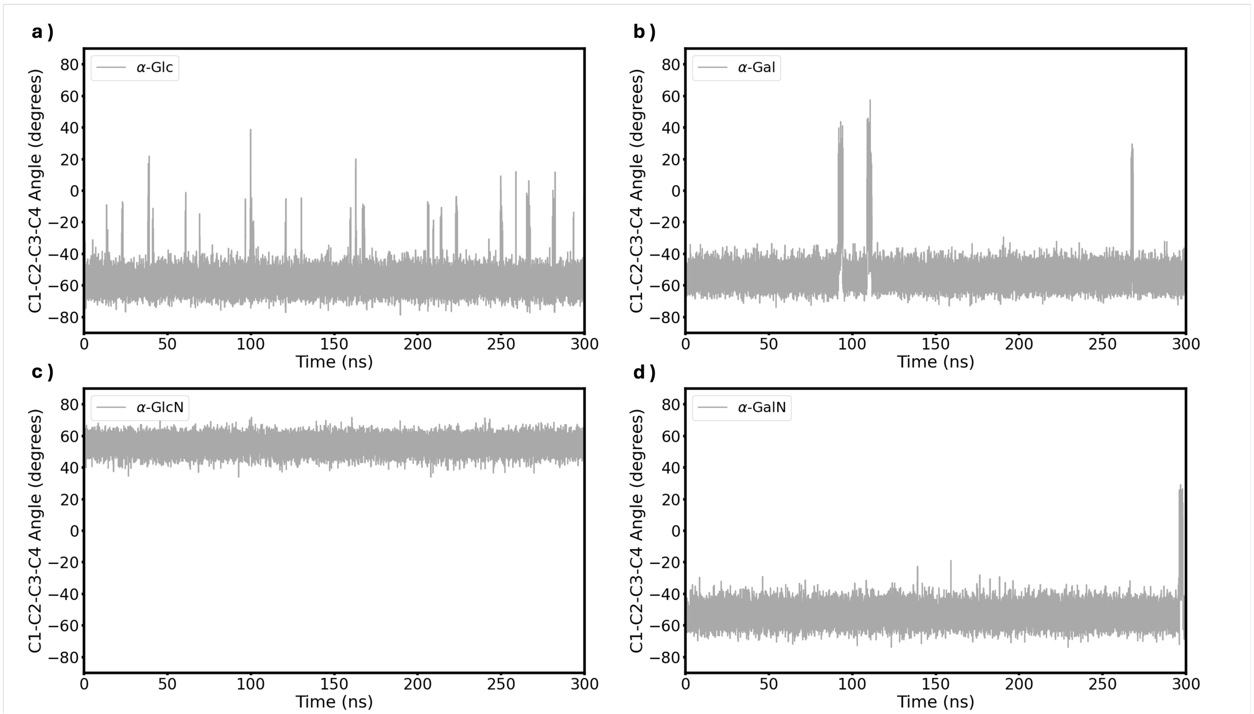

Figure S12: Time evolution of the torsion angle $C_1 - C_2 - C_3 - C_4$ in $\alpha-$Glc, GlcN, Gal, and GalN during 300 ns, second replicate. $C_1 - C_2 - C_3 - C_4$ is -55° when in chair conformation. a) α-Glc, b) α-GlcN, c) α-GlcNAc, d) α-Gal. Overall, α-Glc, α-GlcN, α-Gal, and α-GalN spends 1.7, 0.01, 2.0, 0.8% of the time, respectively, in a conformation other than chair.
